# A Mini Review of Functional Hydrogels for the Treatment of Oral Diseases: Opportunities and Future Prospects

**DOI:** 10.3390/polym18182255

**Published:** 2026-09-16

**Authors:** Huixu Li, Xinru Feng, Pingping Bao, Jing Shen, Ding Zhang

**Affiliations:** 1 Department of Endodontics, Tianjin Stomatological Hospital, School of Medicine, Nankai University, Tianjin 300041, China; 2 Tianjin Key Laboratory of Oral and Maxillofacial Function Reconstruction, Stomatology Institute of Nankai University, Tianjin 300041, China; 3 International Dental Clinic, Tianjin Stomatological Hospital, School of Medicine, Nankai University, Tianjin 300041, China; 4 School of Materials Science and Engineering, Nankai University, Tianjin 300350, China; zhangding@nankai.edu.cn

**Keywords:** functional hydrogels, oral diseases, tooth diseases

## Abstract

Oral health represents a cornerstone of systemic health. Driven by diverse dietary structures and lifestyle habits, nearly everyone is affected by common oral diseases, including oral ulcers, tooth discoloration, and dental caries, etc. However, limited by the inherent properties of conventional oral biomaterials, existing clinical treatments still suffer from insufficient reliability and stability, leaving substantial clinical demands unmet. In recent years, functional hydrogels have aroused widespread interest in the biomedical field owing to their favorable biocompatibility, biodegradability, and tunable mechanical properties, exhibiting promising application potential in oral disease treatment. This paper reviews the recent progress of functional hydrogels in the treatment of oral diseases, with emphasis on their working mechanisms and therapeutic outcomes. Lastly, the current challenges and future opportunities of functional hydrogels in oral clinical applications are also critically discussed. This work is expected to provide valuable guidance for the design and development of innovative therapeutic strategies for oral diseases.

## 1. Introduction

Oral health serves as the foundation of national health [1,2,3]. However, due to factors such as unreasonable dietary structure and poor oral hygiene habits, oral health problems in the general population have become increasingly severe [4,5]. The tooth discoloration, dental caries, and oral ulcers are among the most common oral diseases [6,7,8,9,10,11]. At present, the efficacy of oral disease treatment is still limited by existing oral medical materials. For instance, the restorative materials used for dental caries may degrade or even detach over time, resulting in treatment failure. In addition, conventional therapeutic methods often suffer from low drug delivery efficiency and short residence time in the oral cavity, leading to unsatisfactory outcomes [12,13,14]. Therefore, the development of high-performance oral medical materials is crucial for improving the reliability and stability of oral disease treatment.

**Figure 1:**
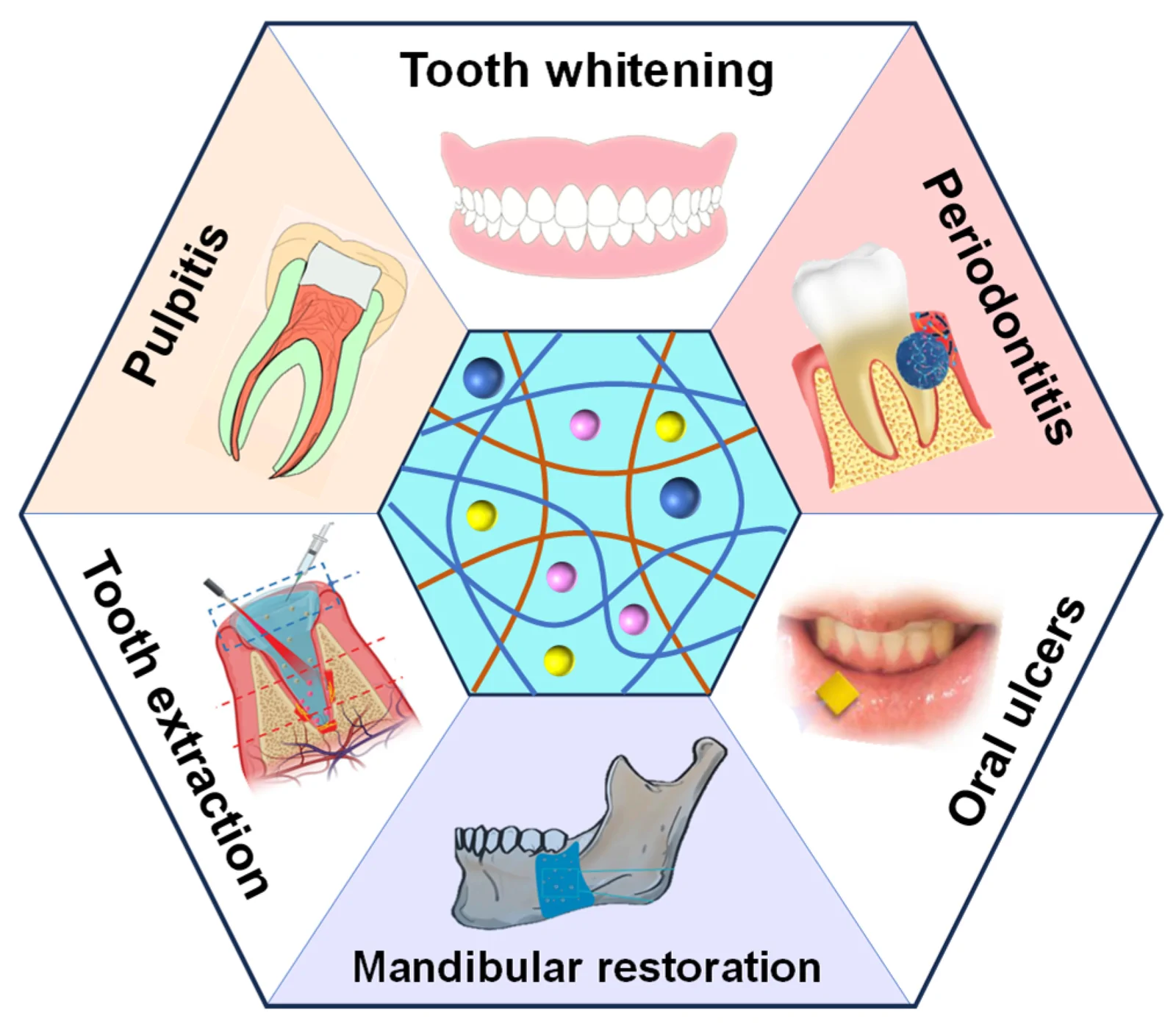
Functional hydrogels for the treatment of common oral diseases. Reproduced with permission from [7], Copyright 2022, American Chemical Society. Reproduced with permission from [23], Copyright 2023, MDPI. Reproduced with permission from [28], Copyright 2023, Wiley. Reproduced with permission from [29], Copyright 2023, Elsevier. Reproduced with permission from [30], Copyright 2021, American Chemical Society.

Functional hydrogels are water-based polymeric materials with a three-dimensionally cross-linked network structure and tailored functionalities, which can absorb and retain large amounts of water without dissolving. Their physical state lies between solids and liquids: they can maintain shape and volume under certain conditions while enabling solute diffusion and permeation. Functional hydrogels exhibit superior water absorption and retention, favorable biocompatibility and biodegradability, stimuli responsiveness, self-healing ability, and tunable mechanical properties [15,16,17,18,19,20,21]. To date, functional hydrogels have been widely used in biomedicine, serving as wound dressings to maintain moisture and accelerate tissue repair and also as drug delivery systems to enhance therapeutic efficacy by controlling drug release rate. Owing to their unique physicochemical properties and excellent biocompatibility, functional hydrogels represent ideal candidates to address challenges in the complex oral environment [22,23,24,25,26,27]. As shown in Figure 1, they have demonstrated great application potential and promising therapeutic efficacy in the treatment of various oral diseases, including dental caries, periodontitis, periapical disease, oral mucosa, and oral cancers [7,23,28,29,30].

Several review articles have summarized the research progress of functional hydrogels in the field of oral diseases; however, most of them focus only on a part of oral diseases and do not cover the full spectrum, such as concentrating solely on dental diseases [2], periodontitis [14], or oral tissue regeneration [23]. This paper aims to comprehensively outline the recent advances of functional hydrogels in the treatment of oral diseases. First, we introduce the classification and characteristic properties of functional hydrogels. Then, the working mechanisms and therapeutic efficacies of functional hydrogels in treating various oral diseases are highlighted and analyzed. Finally, the challenges, opportunities, and future development directions of functional hydrogels in clinical oral applications are discussed.

## 2. Classification of Functional Hydrogels

Functional hydrogels can be classified based on their sources (natural, synthetic, or hybrid), crosslinking mechanisms, and stimulus responsiveness [31,32]. By using different types of functionalization design methods, the characteristics of functional hydrogels (such as drug release properties, adhesion) can be optimized and regulated, thereby enabling the design and synthesis of functional hydrogels for the treatment of various oral diseases according to the requirements of use [33].

### 2.1. Classification by the Source

Natural hydrogels are derived from naturally occurring polymers, including proteins—such as collagen, elastin, fibrin, gelatin, and silk fibroin—and polysaccharides, such as chitosan, alginate, and glycosaminoglycans [31,32]. These materials generally exhibit excellent biocompatibility, biodegradability, and low immunogenicity; however, their mechanical strength is often relatively limited [32,34]. For example, cellulose-based hydrogels can be fabricated from non-woody biomass, providing a sustainable alternative for hydrogel development [35]. In addition, their application of natural hydrogels in load-bearing sites (e.g., periodontal repair) is severely limited by poor mechanical strength. By using structural engineering strategies, such as optimizing crosslinking density and blending specific tri-polymer matrices, the mechanical properties can be adjusted according to the practical requirements [36].

Synthetic hydrogels are prepared from synthetic polymers, such as poly(acrylic acid), poly(vinyl alcohol), and polyacrylamide [31]. A key advantage of synthetic hydrogels lies in the precise tunability of their physicochemical properties, and they typically exhibit higher mechanical strength than natural hydrogels [32].

Hybrid hydrogels combine the advantages of both natural and synthetic polymers, aiming to overcome the inherent limitations of individual hydrogel types. For instance, they integrate the bioactivity of natural polymers with the enhanced mechanical robustness of synthetic counterparts [32].

Natural hydrogels are more suitable for low-stress soft-tissue repair scenarios such as oral mucosa regeneration. Nevertheless, their intrinsic mechanical deficiencies limit their applications in high-load sites like periodontal bone regions. Synthetic hydrogels possess tunable physicochemical properties but lack inherent biological activities, which generally necessitates incorporation of bioactive factors to enhance their tissue-inductive capacity. Hybrid hydrogels achieve balanced material performances and have emerged as a mainstream research direction for oral-applicable hydrogels. Nevertheless, intricate fabrication procedures and unsatisfactory long-term in vivo safety remain critical bottlenecks hindering their clinical translation.

### 2.2. Classification by Crosslinking Mechanism

Physically crosslinked hydrogels are formed through noncovalent interactions that induce polymer chain entanglement and network formation, without the generation of new covalent bonds. These interactions are typically reversible. Representative fabrication strategies include physical blending, freeze–thaw cycling, ultraviolet-induced noncovalent assembly, and supramolecular self-assembly.

Chemically crosslinked hydrogels are fabricated via covalent bonding between polymer chains to form three-dimensional networks. Owing to the high bond energy of covalent linkages, the resulting crosslinking is generally irreversible unless chemical bond cleavage occurs. Common crosslinking strategies include chemically initiated free-radical polymerization (e.g., ammonium persulfate initiation), photo-initiated covalent polymerization, Schiff’s base reactions, and epoxy ring-opening reactions.

### 2.3. Classification by Responsiveness

Stimuli-responsive hydrogels (also known as smart hydrogels) can undergo changes in structure or properties in response to external or internal stimuli, such as pH, temperature, light, electric fields, magnetic fields, and enzymes [13,37,38,39]. This “smart” behavior enables on-demand drug release and targeted delivery, making these hydrogels particularly attractive for advanced biomedical applications [13,37]. It is noted that diverse stimulus modalities exhibit distinct performances within the oral cavity. pH-responsive hydrogels can respond to pH shifts at inflamed oral lesions without auxiliary equipment; however, dynamically fluctuating oral pH modulated by food intake and salivary buffering frequently triggers premature non-specific drug release, compromising controllability. Thermo-responsive hydrogels achieve sol–gel transition relying on physiological body temperature with facile operation, yet limited local temperature variation inside the oral cavity fails to generate sufficient responsive gradients, leading to unsatisfactory lesion-specific recognition. Light-responsive hydrogels realize precisely tuned release by adjusting illumination parameters, benefiting from high controllability; nevertheless, limited light penetration depth caused by tissue obstruction restricts their practical implementation inside the oral cavity.

Non-stimuli-responsive hydrogels maintain relatively stable properties under specific environmental conditions, with drug release primarily governed by diffusion and matrix degradation mechanisms.

## 3. Characteristics of Functional Hydrogels and Their Advantages in Oral Treatment

Hydrogels are three-dimensional network structures formed by hydrophilic polymers through physical or chemical crosslinking, enabling them to absorb and retain large amounts of water and thereby exhibit soft-tissue-like properties [15,31,40]. These characteristics endow hydrogels with excellent biocompatibility, flexibility, and permeability, making them highly attractive materials in the biomedical field, particularly for drug delivery and tissue engineering applications [31,40,41].

**Figure 2:**
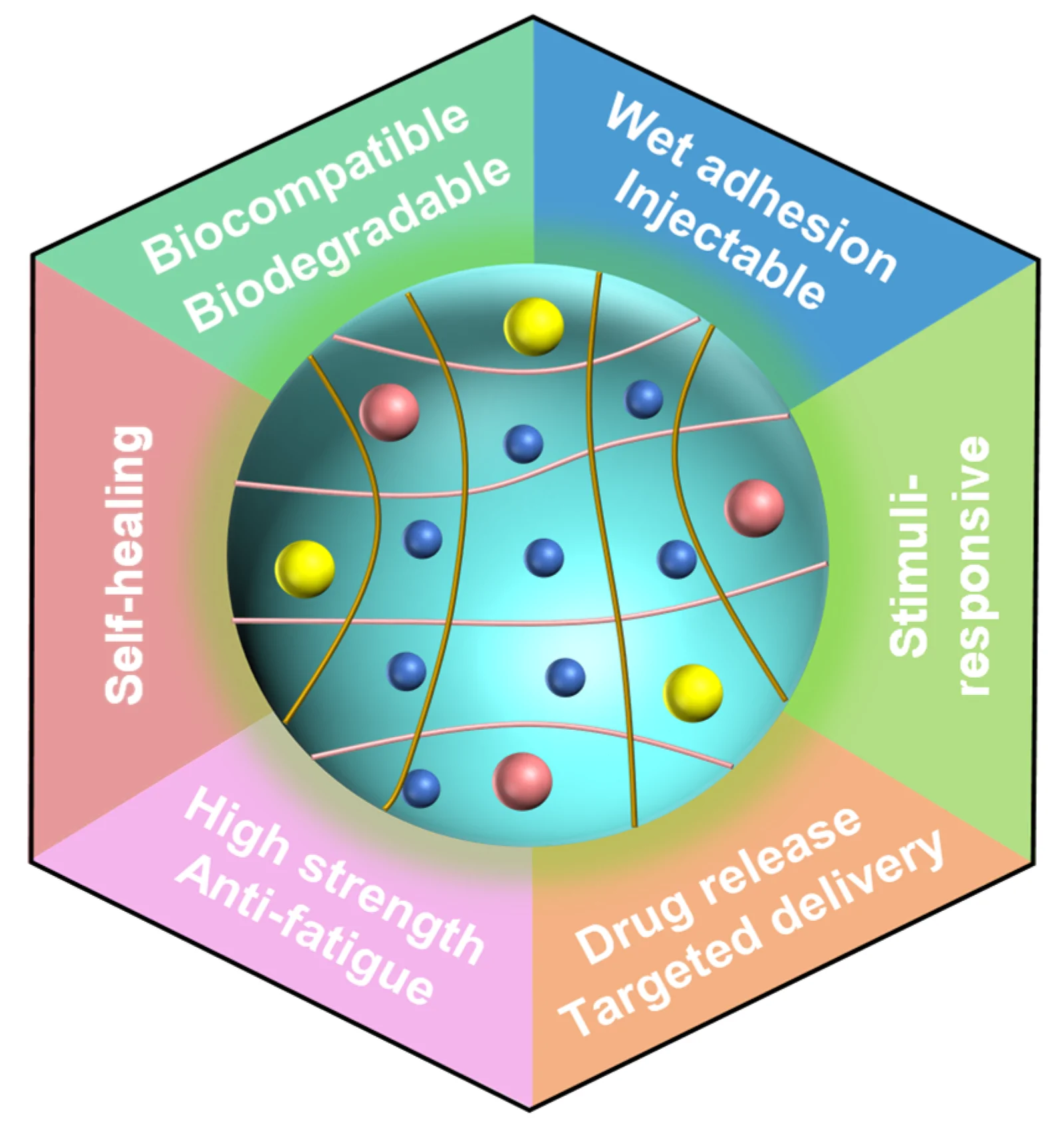
The characteristics and advantages of functional hydrogels.

Figure 2 summarizes the common properties and characteristics of functional hydrogels, and the advantages of functional hydrogels are especially pronounced within the oral environment.

**Biocompatibility**: The oral cavity represents a complex biological environment, which imposes extremely high requirements on the biocompatibility of medical materials. Hydrogels are generally fabricated from natural polymers (e.g., collagen, chitosan, alginate, hyaluronic acid) or synthetic polymers (e.g., polyacrylic acid, polyvinyl alcohol). Among them, natural polymer-based hydrogels are particularly favored owing to their excellent biocompatibility and biodegradability [32,34,42]. For instance, chitosan exhibits great potential in periodontal therapy due to its antibacterial properties and favorable biocompatibility [43].

**Tunable physicochemical properties**: The porosity, swelling behavior, degradation rate, and mechanical properties of hydrogels can be precisely regulated by adjusting the polymer composition, crosslinking strategy, and fabrication processes [44,45]. This tunability enables hydrogels to meet the specific requirements of different intraoral sites. For instance, load-bearing regions for periodontal tissue repair may require hydrogels with higher mechanical strength [15,32].

**Sustained drug release and targeted delivery**: The unique moist environment of the oral cavity and continuous salivary flow often make it difficult to maintain effective local drug concentrations. Hydrogels can encapsulate drugs within their three-dimensional networks to achieve sustained, controlled, and targeted drug release, thereby improving local drug bioavailability and therapeutic efficacy [43,46,47,48]. For example, injectable hydrogels can deliver drugs or bioactive molecules to lesion sites via minimally invasive approaches [43,49].

**Stimuli responsiveness**: Stimuli-responsive smart hydrogels can alter their physicochemical properties in response to external or internal stimuli (temperature, pH, light, enzymes, redox potential, magnetic fields), thereby enabling on-demand drug release [32,38,39,40,41,42,43,44,45,46,47,48,49,50]. Such intelligent characteristics endow hydrogels with significant advantages in the precise treatment of oral diseases. Stimuli-responsive hydrogels hold significant application value in root canal disinfection, pulp inflammation management, and periapical tissue regeneration [13,37]. Their ability to controllably release therapeutic agents helps improve treatment precision, enhance therapeutic efficacy, and minimize side effects [37].

## 4. Applications of Functional Hydrogels in Oral Disease Treatment

Functional hydrogels have been widely used in the treatment of various oral diseases and in the field of oral tissue engineering [22,23,44,50,51]. These include aspects such as periodontal diseases, oral mucosal diseases, dental and pulp diseases, as well as maxillofacial tissue regeneration.

### 4.1. Periodontal Diseases

Periodontitis is a chronic inflammatory disease caused by bacterial biofilms and host immune responses, leading to the destruction of periodontal tissues including gingiva, periodontal ligament, and alveolar bone [14,52]. Hydrogels can serve as drug delivery systems for the sustained release of antibacterial agents, anti-inflammatory drugs, antioxidants, and tissue regeneration-promoting factors, thereby combating pathogenic bacteria, alleviating inflammatory responses, and facilitating periodontal tissue repair [52,53,54]. For instance, polydopamine-based hydrogels exhibit considerable potential in the prevention and treatment of periodontal diseases owing to their versatile properties [55]. Hyaluronic acid-based hydrogels can be employed to encapsulate mesenchymal stem cells, providing a favorable microenvironment for periodontal tissue regeneration [16,56].

**Figure 3:**
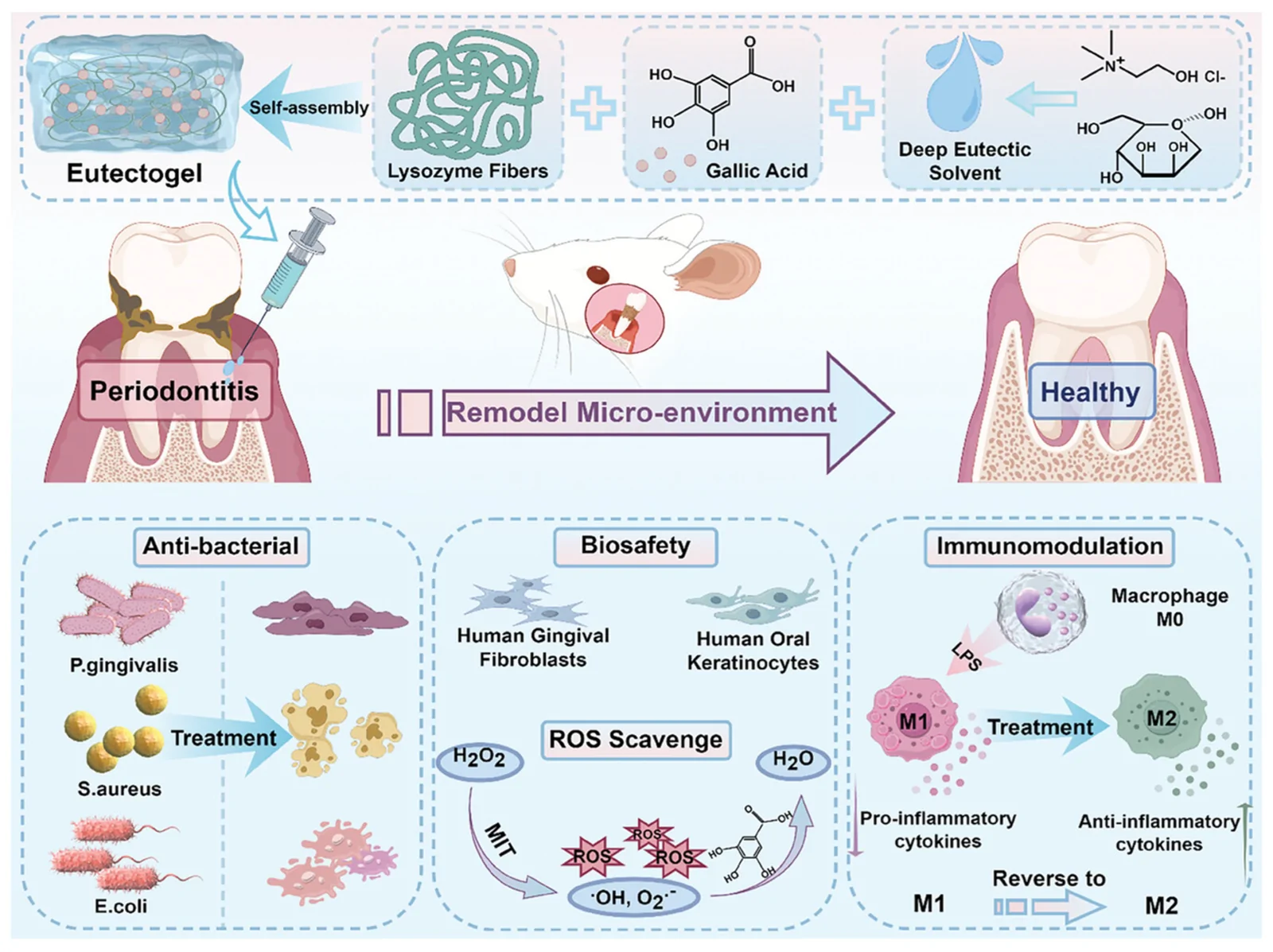
The self-assembly eutectogel for treating chronic periodontitis (MIT: mitochondria). Reproduced with permission from [57], Copyright 2024, Wiley.

As shown in Figure 3, an injectable eutectic hydrogel was fabricated via self-assembly of gallic acid (GA), fibrillated lysozyme (FLy) and deep eutectic solvent (DES) [57]. The hydrogel achieves nearly 100% bactericidal activity against tested strains and exhibits excellent immunomodulatory effects. In a rat chronic periodontitis model, it markedly relieves local inflammation and inhibits alveolar bone loss, owing to DES-mediated transmembrane release of gallic acid. This self-assembled eutectic hydrogel shows great potential for inflammatory disease therapy, enabling bacterial eradication, ROS scavenging and macrophage regulation by enhancing the cellular penetration of small-molecule drugs.

### 4.2. Dental Caries Treatment and Tooth Whitening

Dental caries is one of the most prevalent chronic bacterial diseases worldwide, resulting in the demineralization of dental enamel and dentin [58,59]. Hydrogels can be used to treat dental caries through antibacterial effects and remineralization. Hydrogels loaded with antimicrobial agents can act directly on carious lesions, inhibit the growth of cariogenic bacteria, control biofilm formation, promote tooth remineralization, and repair early carious damage [58,59].

**Figure 4:**
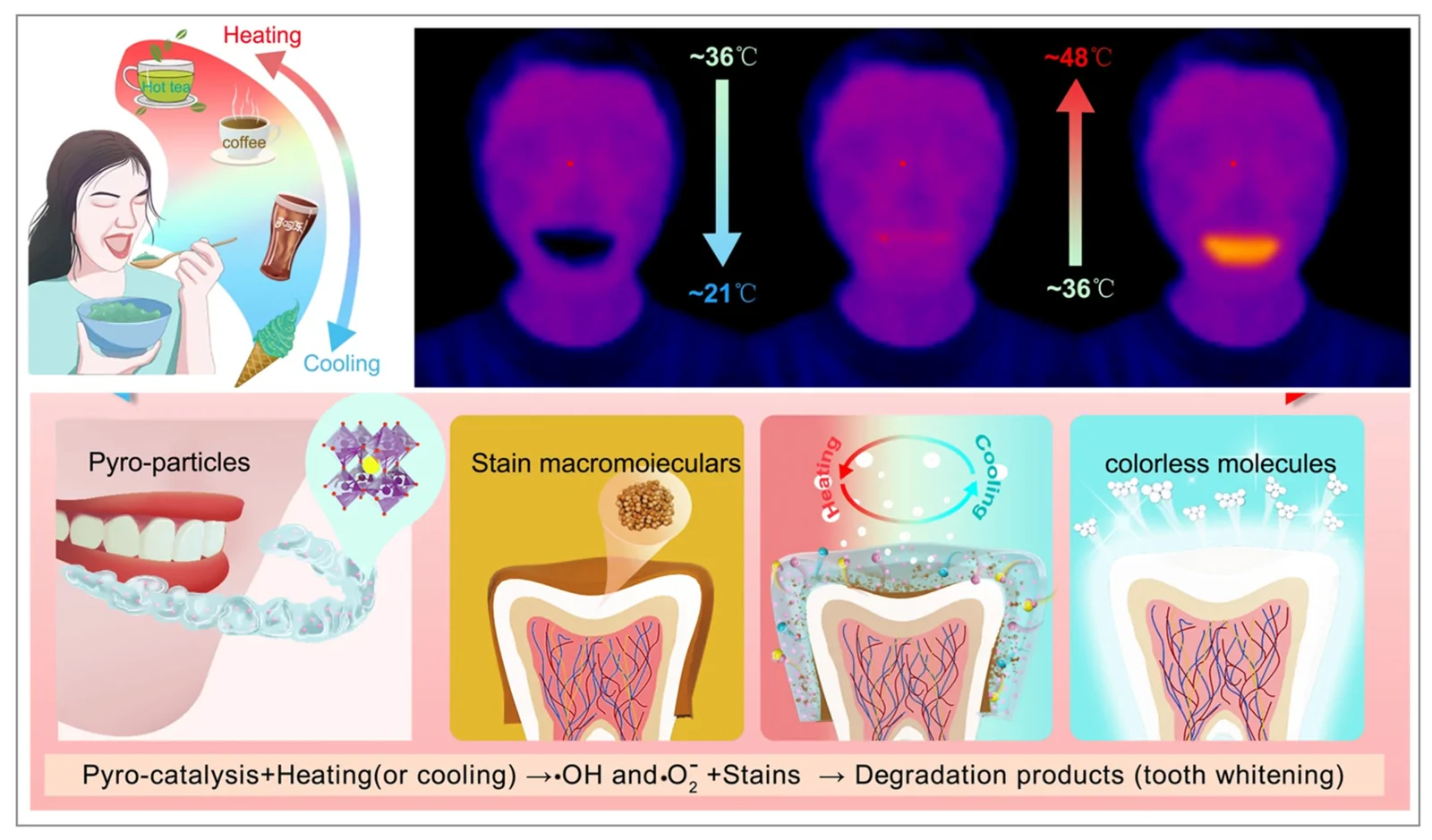
Pyroelectric hydrogel for tooth whitening through periodic changes in oral temperature. Reproduced with permission from [60], Copyright 2022, Springer Nature.

By loading pyroelectric nanoparticles into hydrogels and applying the pyroelectric hydrogels onto tooth surfaces, the polarization intensity of the pyroelectric particles can be altered by temperature fluctuations in the oral cavity, thereby inducing the generation of reactive oxygen species (ROS). As shown in Figure 4, these ROS can degrade large pigment molecules on tooth surfaces to achieve tooth whitening [60]. Furthermore, researchers incorporated piezoelectric nanoparticles into hydrogels to create piezoelectric hydrogels. When these hydrogels are attached to the surface of teeth, by utilizing an ultrasonic-induced piezoelectric effect, the piezoelectric hydrogels can also achieve teeth whitening by generating ROS [61].

### 4.3. Oral Mucosal Treatment and Tissue Engineering

Oral mucosal diseases often cause pain, as well as difficulties in speech and feeding [42]. Functional hydrogels play a positive role in promoting oral and maxillofacial wound healing, exhibiting unique advantages especially in hemostasis, anti-inflammation, accelerating cell proliferation and tissue remodeling [39]. Biomacromolecule-based hydrogels, including those derived from chitosan, hyaluronic acid, alginate and gelatin, are emerging as novel therapeutic options for oral mucosal lesions due to their excellent biocompatibility and advantages in local drug delivery [13]. However, the complex oral environment (humidity and continuous movement) often leads to a low adhesion between the hydrogel and biological tissues, shortening the duration of drug retention, thereby resulting in low drug utilization and poor therapeutic effects.

**Figure 5:**
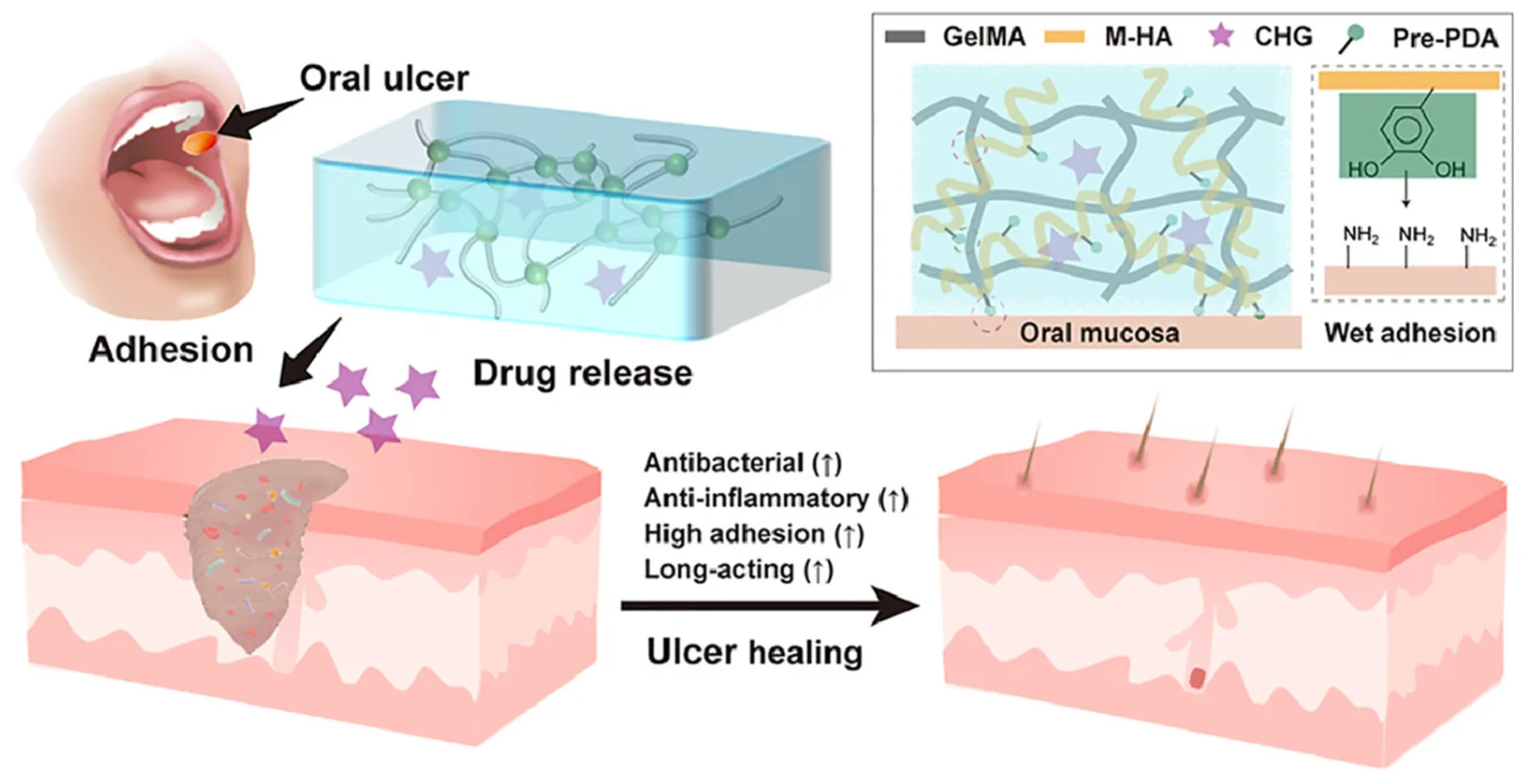
A drug-loaded hydrogel with good adhesion properties used for oral ulcer treatment. Reproduced with permission from [62], Copyright 2024, Elsevier.

Researchers designed a mussel-mimetic adhesive hydrogel with an interpenetrating polymer network (IPN) structure for treating oral ulcers [62]. As shown in Figure 5, the hydrogel was constructed using dopamine-modified hyaluronic acid (M-HA) and UV-crosslinked gelatin methacryloyl (GelMA) and loaded with chlorhexidine gluconate (CHG) to achieve sustained drug release and improved therapeutic outcomes. The GelMA/M-HA interpenetrating network hydrogel exhibits outstanding wet-tissue adhesion, which originates from mussel-chemistry-inspired polydopamine-prepolymerized M-HA. Oxidative prepolymerization within catechol-grafted hyaluronic acid (C-HA) produces M-HA rich in catechol-quinone moieties. Under wet conditions, quinone groups form covalent bonds with amine and thiol groups on mucosal proteins and displace the interfacial hydration layer via multiple noncovalent interactions such as hydrogen bonding and π-π stacking. Meanwhile, the interpenetrating network formed between M-HA and photocrosslinked GelMA provides sufficient bulk cohesion through chain entanglement and physical crosslinking. The relatively low modulus of GelMA/M-HA enables favorable mechanical matching with soft oral mucosa, thereby endowing the hydrogel with superior adhesive performance in the humid oral environment [62]. Benefiting from excellent wet-tissue adhesion, favorable biocompatibility, and combined antibacterial and anti-inflammatory effects, this hydrogel significantly promoted the healing of oral ulcers and infected wounds in rats, representing a reliable and promising candidate for clinical oral ulcer treatment.

### 4.4. Tooth Extraction and Implantation Treatment

When teeth need to be extracted, the healing of the extraction socket is also very important. Functional hydrogels, due to their excellent adhesion, injectability and plasticity, are ideal materials for promoting the healing of the extraction socket [28,63]. Through drug loading design, functional hydrogels can further achieve antibacterial, disinfection and hemostasis effects. In addition, functional hydrogels also have degradability characteristics. They gradually degrade as the biological tissue regenerates, thereby achieving effective wound healing. Functional hydrogels can also be used to assist in dental implantation, providing a safe and sterile environment for the implant and reducing HRP penetration, improving the effectiveness and reliability of dental implantation.

**Figure 6:**
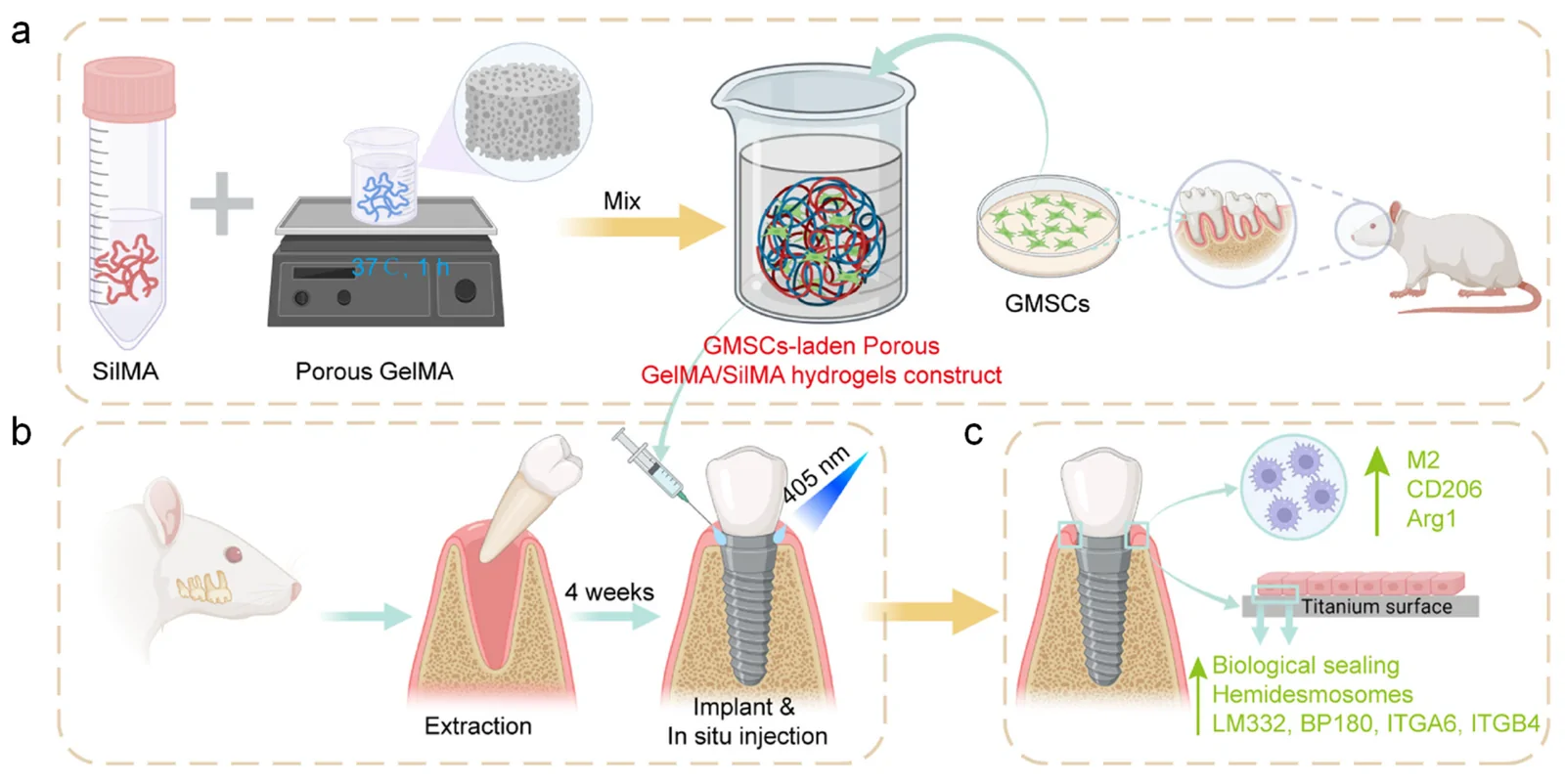
Injectable photocrosslinking GelMA/SilMA hydrogel encapsulated GMSCs for tooth implantation treatment. (**a**) Synthesis schematic diagram of the GelMA/SilMA hydrogel. (**b**) Application of the hydrogel in a rat early implant placement model. (**c**) Multiple synergies of the hydrogel in the peri-implant epithelium (PIE) integration. Reproduced with permission from [64], Copyright 2022, Elsevier.

Researchers successfully prepared injectable photocrosslinking porous gelatin methacryloyl (GelMA)/silk fibroin glycidyl methacrylate (SilMA) composite hydrogel [64]. By loading gingival mesenchymal stem cells (GMSCs), it can be used for the repair of epithelial closure around implants (Figure 6). This hydrogel significantly enhances the in vitro survival, proliferation and spreading of GMSCs, upregulates the expression of desmosome-related genes/proteins, promotes M2-type polarization of macrophages and inhibits M1-type polarization. The early implant model in rats confirmed that it can enhance the biological sealing of the interface between the implant and epithelium, reducing HRP penetration, providing a new combined treatment strategy of stem cells–hydrogel for the prevention of peri-implantitis. Notably, in this GMSC-laden porous GelMA/SilMA system, physical parameters of the hydrogel mesh such as porosity do not directly reprogram macrophages via mechanotransduction. Instead, they indirectly mediate the phenotypic switch from pro-inflammatory M1 to pro-healing M2 macrophages by constructing a permissive three-dimensional microenvironment for encapsulated GMSCs. The optimized P/S6 formulation possesses interconnected pores that support high viability, spreading and proliferation of GMSCs. This porous 3D niche amplifies the paracrine secretion of immunomodulatory soluble mediators by GMSCs. These stem-cell-derived factors further downregulate M1 markers and upregulate M2 markers, driving macrophage phenotypic transition.

In addition, functional hydrogels can also mimic the structure of the natural extracellular matrix, providing scaffolds for cell adhesion, proliferation, and differentiation, and promoting the regeneration and repair of damaged oral tissues [16,23,43]. This is crucial for the restoration of periodontal tissue, dental pulp tissue, and jawbone defects. For example, cellulose-based biomaterials exhibit favorable functions in promoting oral tissue regeneration and cell proliferation [65].

Although numerous laboratory investigations have demonstrated the therapeutic potential of oral hydrogels, few advanced-function hydrogels have completed regulatory authorization and commercialization. For instance, Episil^®^ is an in situ-forming mucoadhesive gel approved in China, the United States and Europe, which relieves pain from chemo-/radiotherapy-induced oral mucositis merely via physical protection without pharmacologically active ingredients. However, most hydrogels remain confined to in vitro and small-animal studies. Multiple barriers hinder their translation toward commercial products, including rigorous medical-device regulatory requirements, maintaining stable performance under dynamic oral conditions with salivary flushing, mastication and complex microbiota, scalable manufacturing, residual-substance control, and comprehensive long-term in vivo biosafety assessment. These gaps highlight the substantial divide between promising lab-scale outcomes and clinically deployable products.

The therapeutic performance of functional hydrogels is heavily governed by intrinsic physicochemical features including swelling capacity, solubility and electrical conductivity. For polycationic biopolymers such as chitosan, cationic–anionic ionic interactions exert dual influences on material stability and antibacterial efficacy. Benefiting from polycationic moieties, these biopolymers can electrostatically attach to negatively charged membranes of *Staphylococcus aureus* and *Escherichia coli*, enabling contact-killing antibacterial effects. Nevertheless, high-ionic-strength salivary fluid may screen surface positive charges and diminish such antibacterial potency. Fortunately, the therapeutic performance of functional hydrogels can be adjusted based on standard manufacturing parameters and baseline engineering benchmarks (such as polymer blending proportion, solvent-casting conditions, swelling ratio, tensile strength, disintegration time) [66].

## 5. Summary

Functional hydrogels have been widely used in the treatment of oral diseases and have exhibited favorable therapeutic efficacy, yet several challenges remain: (I) traditional hydrogels generally possess poor mechanical strength, which limits their application in load-bearing sites such as periodontal tissue repair [15,32]. Developing high-toughness hydrogels by optimizing crosslinking strategies or incorporating reinforcing materials (e.g., nanoparticles) is key to overcoming this limitation [15,32,41]. (II) The degradation rate of hydrogels should match the rate of tissue regeneration. Excessively fast or slow degradation may compromise therapeutic outcomes [23,40]. Meanwhile, the long-term biosafety of hydrogels and their degradation products requires further evaluation [23,40]. (III) Complex oral environmental factors, including pH fluctuations, salivary enzymes, mechanical stress from mastication, and microbial load, impose stringent requirements on the stability and functionality of hydrogels [12,13,14]. It is extremely difficult to truly solve these problems, and this is also a challenge for the future. In general, salivary washing and dilution within the oral cavity can lead to rapid drug loss from hydrogels. This issue can be mitigated by increasing crosslinking density and constructing mucoadhesive interfaces; however, excessively high crosslinking density usually compromises degradability. Repeated mechanical loading from mastication tends to trigger hydrogel failure. Although hybrid strategies can improve mechanical performance, it remains challenging to simultaneously balance flexibility and mucoadhesiveness. Variable oral pH and enzymatic activity accelerate matrix degradation. pH-responsive crosslinking can enhance environmental adaptability, yet it increases the complexity of material fabrication. Biofilms (dental plaques) readily colonize hydrogel surfaces. Incorporation of antibacterial moieties can inhibit biofilm formation, whereas the long-term in vivo cytotoxic risks still require further evaluation. (IV) At present, many hydrogels are still in the preclinical research stage. In-depth investigations are still needed regarding the cost and quality control of large-scale production, as well as the translational process from in vitro studies to in vivo applications and from animal models to clinical practice [51]. In addition, the regulatory approval, manufacturing reproducibility, sterilization methods, storage stability, and cost-effectiveness all require careful consideration. Especially regarding the sterilization methods, this is a crucial issue for biological materials used in the oral cavity. Common sterilization methods may affect the structure, mechanical properties, swelling behavior, and drug release kinetics of hydrogels. This is also a key factor hindering the practical application of functional hydrogels. In the future, this issue needs to be given special consideration.

In the future, research on functional hydrogels in the field of oral disease treatment will focus on the following aspects: (I) developing hydrogels integrating multiple functions such as antibacterial, anti-inflammatory, antioxidant, pro-angiogenic, and tissue regeneration properties to address the complex pathophysiological processes of oral diseases [52,53,54]. (II) Combining 3D printing technology to custom-prepare hydrogel scaffolds with precise shapes and sizes according to the specific lesion sites and needs of patients, thereby achieving precision medicine [60,67]. (III) Further exploring novel stimuli-responsive mechanisms, such as multi-stimuli-responsive hydrogels, to enable more precise control of drug release in response to the dynamic changes in the oral microenvironment. For example, integrating magnetic inorganic nanomaterials into hydrogels enables precise, non-invasive targeted therapy, thermal therapy regulation, or controlled drug release under the influence of an external magnetic field [68]. (IV) Combining hydrogels with stem cells and growth factors to serve as cell delivery carriers and tissue engineering scaffolds, promoting the regeneration of dental pulp, periodontal, and bone tissues [16,56,67]. For instance, in fields such as temporomandibular joint diseases, bone augmentation after maxillary sinus floor elevation, local oral anesthesia, orthodontic tooth movement, periapical lesions, bone engineering, and peri-implant inflammation, stimuli-responsive hydrogels have demonstrated their potential in promoting bone formation, inhibiting inflammation, and achieving precise drug delivery [13]. (V) Numerous existing studies emphasize the favorable biocompatibility of oral hydrogels, whereas potential safety risks derived from these materials are rarely discussed. Incompletely consumed cross-linkers and photoinitiators from chemical or photocrosslinking systems may trigger local cytotoxicity and mucosal irritation. Degradation by-products released from hydrogel matrices can alter local pH microenvironment and provoke mucosal inflammatory responses. For hydrogels incorporated with nanoparticle additives, concerns remain regarding nanoparticle accumulation, cellular internalization and attendant potential cytotoxicity [69,70]. Furthermore, short-term animal assessments cannot fully reflect long-term tissue outcomes, such as chronic inflammation and foreign-body giant-cell reactions [71,72]. Notably, safety evaluations for most oral hydrogels are confined to in vitro cell assays and short-term small-animal models. Sufficient data describing long-term biological performance under realistic oral conditions with salivary flushing, masticatory disturbance and complex microbiota are still lacking, which constitutes a major bottleneck hindering clinical translation.

In summary, functional hydrogels, with their unique biological and physicochemical properties, provide innovative solutions for the prevention and treatment of oral diseases. Through continuous technological innovation and interdisciplinary research, functional hydrogels are expected to play an even more important role in future stomatology, ultimately improving patients’ oral health and quality of life.

## Data Availability

No new data were created or analyzed in this study. Data sharing is not applicable to this article.
